# Supplementary material for: Towards the accurate modelling of antibody−antigen complexes from sequence using machine learning and information-driven docking
Source: Bioinformatics. 2024 Sep 30;40(10):btae583. doi: 10.1093/bioinformatics/btae583 (PMC11483107; doi:10.1093/bioinformatics/btae583)
Supplement: btae583_Supplementary_Data [file btae583_supplementary_data.pdf]

Supplementary Material for ”Towards the accurate modeling of antibody-antigen complexes from sequence using machine learning and data-driven docking”

Marco Giulini[1], Constantin Schneider[2], Daniel Cutting[2], Nikita Desai[2], Charlotte M. Deane[2], Alexandre M.J.J. Bonvin[1]

[1]Bijvoet Centre for Biomolecular Research, Faculty of Science - Chemistry, Utrecht University, Padualaan 8, 3584 Utrecht, CH, The Netherlands

[2]Exscientia plc, The Schroedinger Building, OX4 4GE, Oxford, UK

July 31, 2024

Contents

1 Data set 3

2 On the effect of clustering on docking success rate 5

3 An analysis of medium and high-quality docking poses 6

4 Influence of epitope and paratope RMSD on docking quality 9

5 Epitope and paratope confidence scores as predictors of docking quality 13

6 Energy minimisation of rigid-body solutions increases the top 1 success rate 20

7 Protocol timings 21

References 22

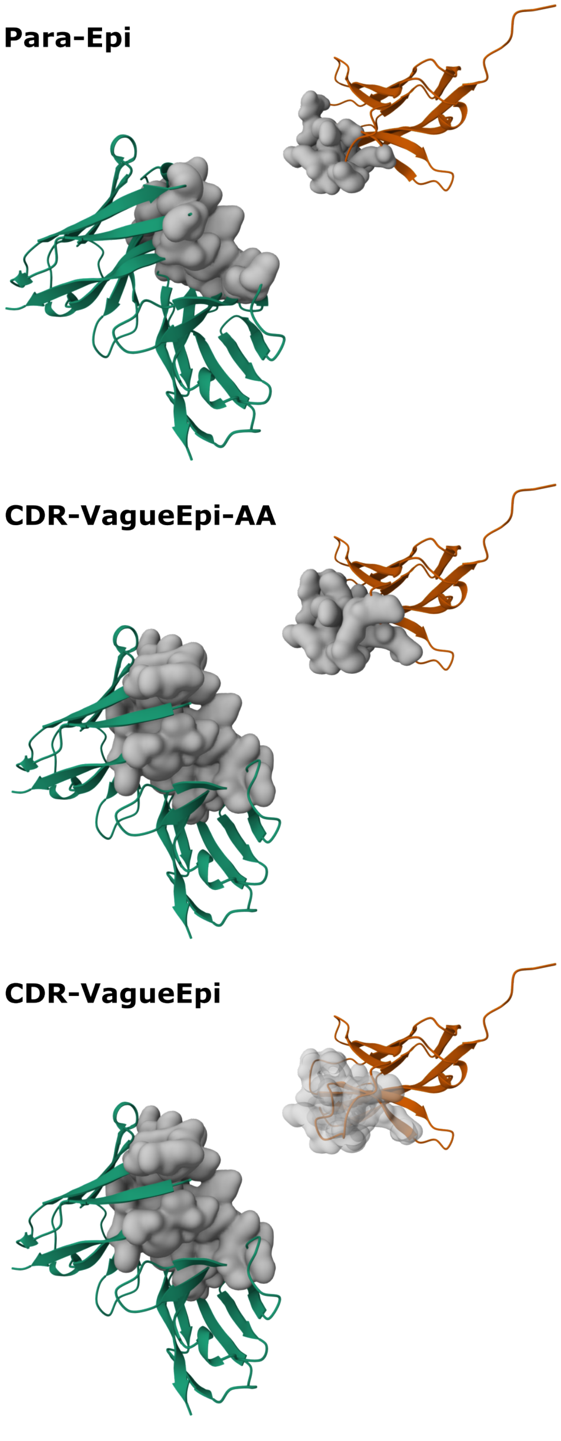

Supplementary Figure 1: Representation of the regions defined as important for the interaction (i.e., converted into ambiguous interaction restraints) in the three scenarios for pdb 7VUX. The (bound) antibody structure is represented in green, while the antigen is coloured in orange. The **Para-Epi** scenario contains information about the true interface, while in the two **CDR-VagueEpi** scenarios all the CDR loops are labeled as potentially active. The only difference between these two scenarios lies in how we treat the vague definition of the epitope for the antigen (labelled as active in **CDR-VagueEpi-AA** and as passive in **CDR-VagueEpi**).

1 Data set

Supplementary Table 1: CDR loop sequences of the 82 antibodies present in the data set

PDB	Heavy Chain	Light Chain	Antigens	H1 Loop	H2 Loop	H3 Loop	L1 Loop	L2 Loop	L3 Loop
7b0b	H	L	F	GFTFTSSA	IVVGSGNT	AAPHCSSTICYDGFDI	QSVSSSY	GAS	QQYGSSPWT
7bbg	H	L	A, C	GGTFSSYA	IIPIFGTA	ARSIELWWGGFDY	QSISSW	DAS	QQYEDYTT
7bbj	H	L	A	GYTFTTYW	IYPGLSDT	ARLLDYAMDY	QDIRSY	YTS	QQGETLPWT
7bnv	H	L	A	GYSFTSYW	IYPGDSDT	ARHPSPYYYGSGSYSGGFDY	QSVSSSY	GAS	QQYDNWPLMHT
7coe	H	L	A	GGTFNNNA	IIPFFGIA	ARDLPRESSYGSGSYYTHYYAMDV	QSVSSSY	DAS	QQYGSSPLT
7daa	H	L	A	GFSLSDYA	IYASGST	ARYYAGSDI	QSISAY	DAS	QTYYAIITYGAA
7dk2	A	B	C	GFTFSSYW	IKQDASEK	ARDLGILWFGDYP	QGISNS	AAS	QQFYSTPRT
7e3o	H	L	R	GFTFSSYA	ISYDGSN	ARGLGLRFLEWPISSY	SSNIGSNT	SNN	AAWDDSLKGV
7e5o	H	L	A	GGSFSGYY	INHSGST	ARGWDLDYYYGMDV	QGIGNY	AAS	LQHGFLPWT
7e72	A	B	E	GYSFTSYW	IHPSDSET	ARGLYGNS	QDIGIS	ATS	LQYASSPYT
7f7e	C	L	E	GFTFSSYA	IVGSGGST	AKSLIYGHYDILTGAYYFDY	QGIGNW	AAS	QQANSFPP
7k7h	H	L	A, C	GYAFTNYK	IDPYNSDS	AGLELTGTLPY	QSLFNSRTRKNH	WAS	KQSHNRALT
7k9j	H	L	A	GYTFTSYW	IYPGSGST	ARWDFYGSRTFDY	QNIGTI	YAS	QQSSSWPLT
7kez	H	L	V	GFNIKDTY	IYPTNGYT	ARGGAVAGTGVYYFDY	QDIPRSISGY	WGS	QQHYTTPPT
7kf0	H	L	C, V	GFNIKDTY	IYPTNGYT	ARGGSFYYYYMDV	QDIPRSISGY	WGS	QQHYTTPPT
7kf1	H	L	V	GFNIKDTY	IYPTNGYT	AKLGIGYYYYGMDV	QDIPRSISGY	WGS	QQHYTTPPT
7kn3	H	L	A	GFTVSLSH	TYGDGNS	AREYYYGMDV	QSVLYSSNNKNY	WAS	QQYYSLPLT
7kn4	H	L	A	GGSLSSVNYY	IYYSGST	ATPGAIMGALHI	SSNIGSNT	FDY	AAWDDSLSAWV
7kql	H	L	T	GGSISSRSYY	IYYSGFT	ATGGPYGDYAHWFEP	QSVSSSY	GAS	QQYGSSPIT
7kyo	H	L	B	GITFSSYA	ISSGGST	ARGPMALLYYRGFDY	QSVDYDGDSY	AAS	QQSNEDPWT
7l0l	H	L	A, B	GGIFRSNA	VVAIFGTT	ARHSGYHITNTFFDY	QSVPSSY	GAS	QQYGSSPYT
7l7d	H	L	E	GFTFMSSA	IVIGSGNT	AAPYCSSISCNDGFDI	QSVSSSY	GAS	QHYGSSRGWT
7l7r	B	A	G	GFTFSSYV	IYRGGST	VKDPKAWLEPEW	QSISKY	AAS	QQSYSNPRT
7l7r	D	C	G	GGSLSSGGYY	IYYSGST	ARDRMDYSGSGVFDY	QSISRW	KAS	QQYGSYSRT
7lf7	A	B	M	GFTFSRCA	ISRDSTYT	ARQIDDYYVDALDY	KIISKY	SGF	QQHNEYPLT
7lfa	B	D	A	GYTFTSHW	IYPGDGDT	ARENLYGYYFDY	TGAVTSGNF	GAD	ALWYSDHWV
7lfb	H	L	X	GYAFTNYG	INTNTGQT	ARLIYDGDYISSDF	ENIYGA	GAT	QNALSMPYT
7lr3	H	L	D	EFTFSDYG	ISSGSNSI	SREAYFAMDY	QDIHKY	YTS	LQYDNLYT
7lr4	H	L	D	DYSLSDYN	INPNHGTT	ASPIHYGNHVPFDY	QDISNY	FTS	QQGITLPWT
7mdj	A	B	C	GYTFTSDW	IIPSYGRA	ARERGDGYFDY	QSIGTD	YAS	QQSNRWPFT
7mrz	X	Y	C	GGSISSSSYY	ISYSGST	ARDSLRYGMDV	QSVLYSSNNKNY	WAS	QQYALAPPRT
7msq	D	E	B	GGSISSYH	IYYSGNT	VREMRRGYSGYDYWDLYAFDI	QGISSY	AAS	QQLNSYPHT
7mzf	H	L	A	EFIVSRNY	IYSGGTT	ARDRGDYLFDY	QSISSW	KAS	QQYNSYFPT
7mzg	H	L	A	GLTVSSNY	FYPGGST	ARDAVYYGMDV	QSISSY	AAS	QESYSTPGLFT
7mzh	H	L	E	GYTFTGYY	INPNSGGT	ARSYYDY	SSNIGHNA	YDD	AAWDDILNGPV
7mzi	H	L	A	GFTFSRFA	ISGSGGST	AKVGWGAFDI	YSNIGSNP	AND	STWDDSLPGPL
7mzj	H	L	A	GFTFSYAW	IKRKSDGGTT	TTDLCRSTSCEHDAFDI	QSIRSY	AAS	QQSYTTPAIT
7mzk	N	M	B	GYTFTSYY	INPSGGGT	AKDRVTIFWGNGMDV	QSVLYSSNNKNY	WAS	HQYSTTPLT
7mzm	H	L	A	GFTFSDYY	ISGGSSFT	ARSPPTGDSSDWYDSPAYNNYYMDV	QSVSSSY	GAS	QQYGTSPTVT
7n3i	H	L	C	GFTVSSNY	IYSGGST	ARDLYSSGGTDI	QSVSSSY	GAS	QQYGSSPGT
7n4i	H	L	C	GYSFISYW	IYPGDSDT	ARLLYYSDSSPLDS	QSISTY	AAS	QQSHSTPRT
7n4j	H	L	A	GDSISSSDYS	IYYIKNT	ARERPPFDVVVVPAARPYNWFDP	SSNIGAGYD	GNN	QSYDSSLSGSKV
7np1	H	L	A	GITVSSNY	IYSGGST	ARGEGGSIVGVTSDY	QSISRY	AAS	QQSYSTLPYT
7nx3	B	C	F	GYAFSSYW	IYPGDGDT	ARSRGYFYGSTYDS	ESVDNYGISF	AAS	QQSKEVPWT
7phu	B	C	A	GYTFTDYY	INPNSGGT	ARDLWFGESPPYYGVDV	NIGSYS	YDS	QVWDTNTDHVV
7phw	B	C	A	GFDFSSYA	IRNDGSFT	TKSADDGGHYSDFSGEIDA	TYNY	YND	GNSDSRNVA
7pi7	B	C	A	GFNIKDTY	IDPANGNT	ARDVLYFDV	ESVDSYGNSF	RAS	QQSNEDRT
7pr0	H	L	E	GFTFSSYS	ISSSSSTI	ASPGGITAAGTSVLFGYYGMDV	QSLLHSNGYNY	LGS	MQALQTPITWT
7ps0	H	L	E	DGSISSSDYY	IYYTGST	ARLVVPSPKGSWFDP	SIDVGNYNL	EGS	CSYVGSSTYV
7ps1	A	B	E	GLTVRSNY	IYSGGST	ARDLVVYGMDV	QSVSSSS	GTS	QQYGSSPL

Continued on next page

Supplementary Table 1: CDR loop sequences of the 82 antibodies present in the data set (Continued)

7ps2	A	B	G	GFTFSNYG	ISYEESNR	AKDQGPATVMVTAIRGAMDV	QSVLYSSNNKNY	WAS	QQYFGSPSIT
7ps2	H	L	G	GHNSPSYW	IDPSDSYT	ARHVVALTHLYPDY	QSVSST	GAS	QQYNNWSTWT
7ps4	H	L	E	GYSFTNYW	IYPGDSGT	ARSRVGATGGYYDYYMDV	SSNLGGNT	SNN	AAWDDSLNGPV
7ps6	H	L	E	GGSITSSNHY	MYYSGST	ARQIGPKRPSQVADWFDP	QGISSY	AAS	QQLNSYPLT
7ps6	C	D	E	GYTFTSYG	ISPYNGNT	ARDGELLGWFDP	SSDVGSYNL	AGS	CSYAGSSTWV
7q0g	A	B	E	GGTFSSSV	IIPLFGSA	AKVSQWALILF	QSVSSSY	GAS	QQYGTSPSWT
7q0g	H	L	E	GFTVSSNY	IYSGGST	ARDHVRPGMNI	QDISNY	DAS	QQYDNLPVT
7q0i	H	L	C	GFTFSSYG	IWYDGSNN	ARSYCSGGFCFGYYYGLDV	NIGTKS	YNS	QVWDSGSDHYV
7qnw	A	B	E	GDSISSSRYY	FYYSGIT	ARPRPPDYYDNSGALLFDI	QSISAW	KAS	QQYISSSPWT
7qny	A	B	E	GFTFDDYA	VSWNSGTI	AREVGGTFGVLISREGGLDY	TIGSKS	DDS	QVWDSSSDRVV
7qu1	A	B	C	GYAFGSHW	IYPGDGDT	ARDDYGTRYYFDY	QDINNY	YTS	QQGKTLPLT
7qu2	A	B	C	GFTFSNYQ	ITVKSDNYGA	SRSGIYDGYYAYAMDY	QIVGTS	WAS	QQYATYPLT
7r89	C	D	B	GFTFSEFF	SRNEANDYTT	ARDAWMGFDY	QEISGY	AAF	LQYASYPCT
7r8l	H	L	E	GITVSSNY	MYAGGST	ARDLYSSGGTDI	QSIGSSY	GAS	QQYGSSPGT
7rah	B	A	E	GFTFSSYG	ISSGGTYT	AREIMRGGGYYFDY	QDISNY	YTS	QQGNTLPYT
7rah	D	C	E	GYAFTNYL	INPGIGNT	ARGLNYGSSQHWYFDV	SSVSSSY	STS	HQYHRSPPT
7rfb	A	B	C	GGTVNT	IFPLLGVP	AKDGVGWSGHGPSQWSGVDV	QSLLHSTGYNY	LGS	MQALEIPRLT
7rk1	C	C	A	GYSITSGYY	ISYDGSN	ATFYDGYDY	QDVSTA	WAS	QQHYSTPFT
7rk2	C	C	A, B	GFSLTSYG	IWSGGST	ARNSLLDAMDY	QSVSNA	YAS	QQDYSSPLT
7rks	H	L	R	GFTFSNYA	ISYDGSNK	ASGYTGYDYFVRGDYYGLDV	SGHSSYA	LNTDGSH	QTWGTGILV
7s0b	A	B	F	GFTFSSYA	ISGSGGST	ARDLWGSGFFAFDV	QDISNY	DAS	QQDAGTPLT
7s11	H	L	C	GLSLTTNS	IWSNGGT	ARNFPYPGINFD	TGAVTTSNY	GTS	SLWYSGHLI
7s13	H	L	C, D	GLSLTNNI	IWSNGGT	ASRDYPGFAY	ELPKRY	EDS	LSTYSDDKLPI
7s4s	H	L	A	GFIVSRNY	IYSGGST	ARDLEVAGAIDY	QSISSSY	GAT	QQYGSSPGYT
7seg	H	L	C	GYTFTSYY	IEPMYGST	ARGSAYYYDFADY	NIGSKN	QDN	QVWDNYSVL
7sem	B	C	F	GFTFSSYS	ISASSSYS	ARARATGYSSITPYFDI	SSNIGAGYD	DNN	QSYDRSLSGV
7shu	E	F	B	GYNITSGYS	VTYDGST	AKGNNYFGHWHFAV	KSVDSDGDSY	AAS	QQSHEDPYT
7shz	K	L	H	GYSITSGYS	IKYSGET	ARGSHYFGHWHFAV	KPVDGEGDSY	AAS	QQSHEDPYT
7si0	I	J	G	GYSITSGYS	VTYDGST	ARGSHYFGHWHFAV	QSVDSDGDSY	AAS	QQSHEDPYT
7soe	B	L	A	GFSVNTGGVG	IYWNDDK	AHVLVWFGEVLPDAFDV	KLGETY	QDN	QAWDKTIAG
7vmu	A	A	B	GVTVSSNY	IYPGGST	ARDLSEKGGMDV	QGISSY	AAS	QQLNSYPR
7vux	H	L	A	GFAFSSYD	ISGGGRYT	ASPYGGYFDV	QSISNF	YAS	QQSNSWPHT

2 On the effect of clustering on docking success rate

As described in the main text, FCC clustering (Rodrigues et al., 2012) is applied at the end of each HADDOCK3 run in order to group together models sharing a consistent fraction of their interfacial contacts. Given the low number of sampled solutions, docking protocols typically give rise to few clusters.

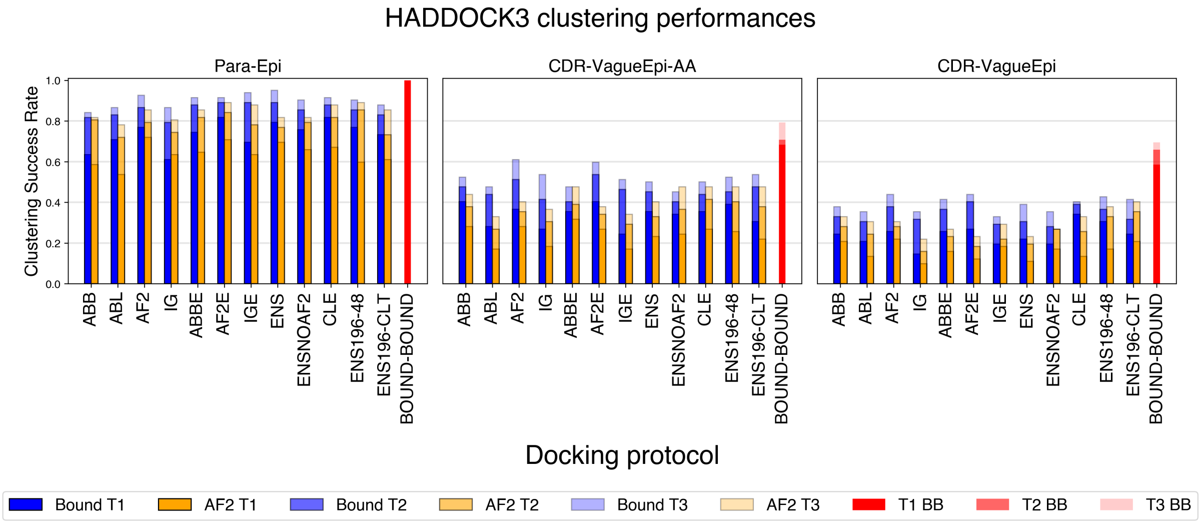

Supplementary Figure 2: Cluster-based success rate (CB-SR) of each protocol upon applying FCC Clustering at the end of the workflow. T1, T2, and T3 bars represent the CB-SR over the first one, two, and three clusters, respectively.

Supplementary Fig. 2 shows the cluster-based success rate (CB-SR) over the top 1, top 2 and top 3 clusters for each protocol. Following Ambrosetti et al. (2020), the CB-SR is calculated as the percentage of times in which at least one of the top four models of the top 1, top 2, or top3 clusters is acceptable or more.

According to this definition, CB-SR is very high for the Para-Epi scenario. More specifically, AF2 performs well, reaching CB-SR of 0.77, 0.87, and 0.93 (resp. 0.72, 0.79 and 0.85) over the top 3 clusters when using the bound (resp. AlphaFold2-modelled) antigen. The AF2 protocol performs better than all the other single tool protocols (ABB, ABL, IG), and reaches an accuracy comparable to the best ensemble protocols. In the lower information scenarios the situation is very similar, with AF2 and AF2E outperforming the other protocols when using the bound antigen. When the unbound structure of the antigen is used, ensemble protocols obtain a slightly higher CB-SR, probably due to the higher diversity in the input structures.

3 An analysis of medium and high-quality docking poses

In the main text we show the statistics on acceptable quality models according to CAPRI criteria, namely structures that contain at least 10 % of the native contacts ($F_{nat} > 0.1$) and possess a value of interface-RMSD (I-RMSD) lower than 4.0 Å or a ligand-RMSD (L-RMSD) below 10 Å.

Supplementary Fig. 3 shows how the success rate when we consider models of at least medium quality ($F_{nat} > 0.3$ and either I-RMSD < 2.0 Å or L-RMSD < 5.0 Å). As expected, the success rate is lower than before, but still HADDOCK3 consistently reaches a T1-med SR values around 0.50 (resp. 0.30) for the bound (resp. AlphaFold2-modelled) antigen in the Para-Epi scenario. In this scenario, the refinement has an important impact on T1-med SR (resp. T10-med SR) for all protocols, with an average improvement of 0.11 (resp. 0.14) when using the bound antigen and of 0.10 (resp. 0.16) for runs targeting an AlphaFold2-generated antigen structure.

In the low-information scenario (CDR-VagueEpi-AA) the number of medium or better quality models is lower, with the best protocols (ABBE and all the ensemble protocols) reaching T10-med SR around 0.40 and 0.28. Here flexible refinement and energy minimisation are less impactful in re-scoring and improving the success rate, displaying a small improvement only considering the T10 SR (0.03).

Supplementary Fig. 4 shows the values of T1-high, T10-high, and T48-high SR, that is, the percentage of times HADDOCK3 generates a high-quality model ($F_{nat} > 0.5$ and either I-RMSD < 1.0 Å or L-RMSD < 1.0 Å) in the top 1, 10, and 48 structures, respectively. High-quality models are hard to reach with the limited sampling present in our protocols (see main text).

In the good knowledge scenario, protocols obtain a good fraction of high-quality models only when employing the bound structure of the antigen. The best protocol in terms of T10-high SR is CLE with 0.27 after refinement. In the CDR-EpiVag-AA scenario (Supplementary Fig. 4b)) most protocols cannot identify more than a handful high-quality poses, with ENS-196-48 and ENS-196-CLT emerging as the best protocols in terms of T10-high SR (0.16).

As in the remaining of the manuscript, also in this case the flexible refinement is very helpful if guided by a good knowledge of the interface (Para-Epi scenario, Supplementary Fig. 4c)), while being detrimental if the restraints are less precise (CDR-VagueEpi-AA scenario, Supplementary Fig. 4d)).

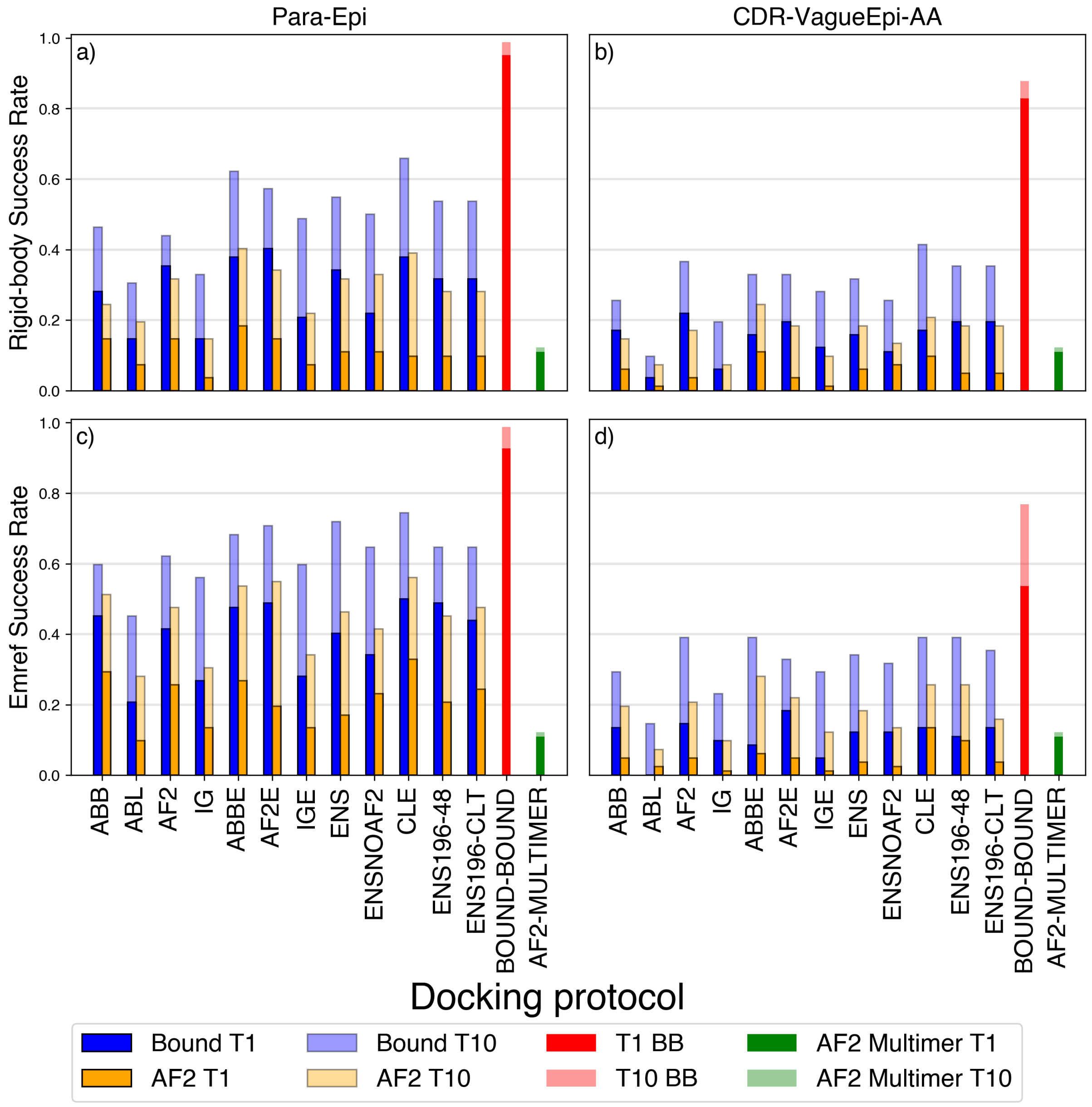

Supplementary Figure 3: Success rates for medium quality poses according to CAPRI criteria.

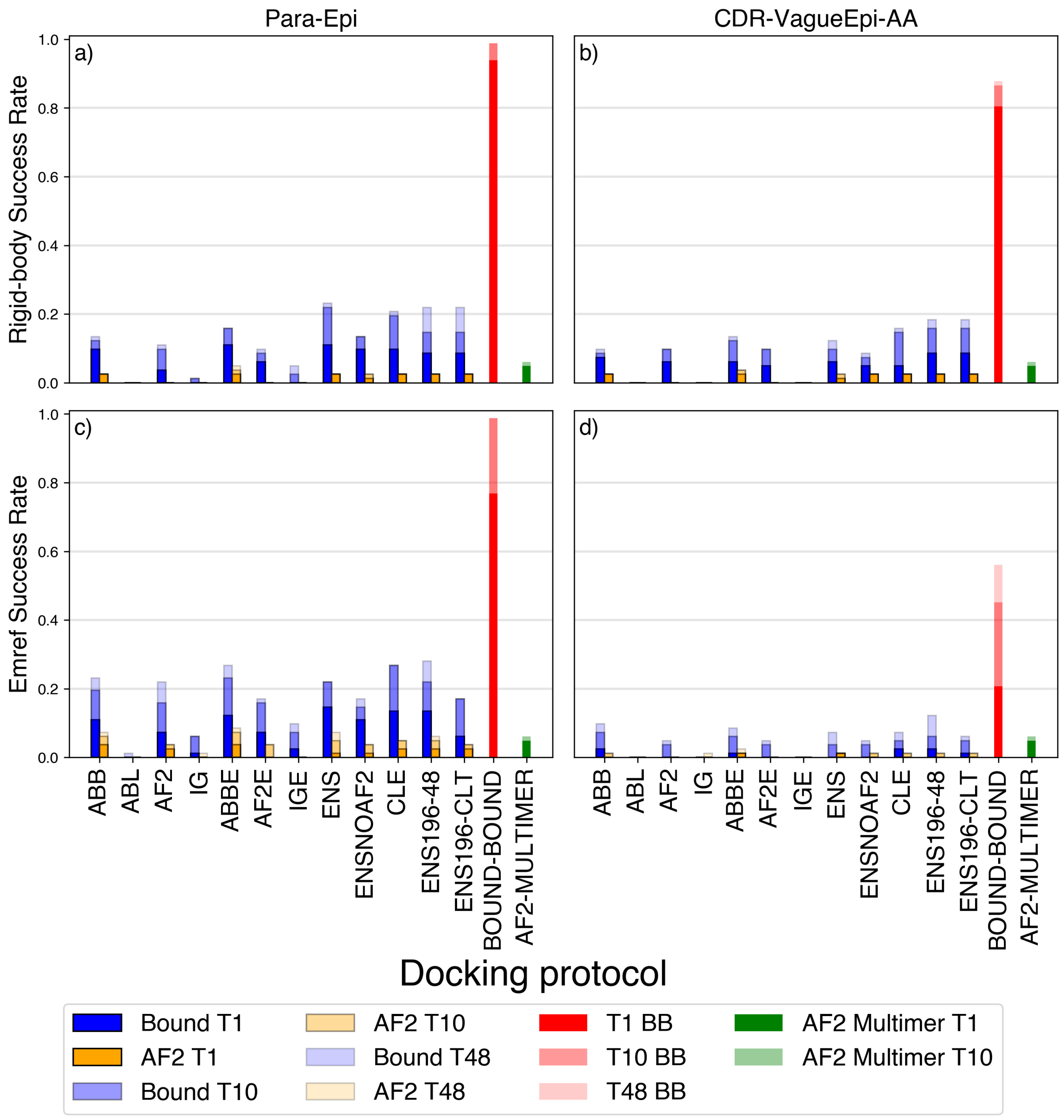

Supplementary Figure 4: Success rates for high-quality poses according to CAPRI criteria.

4 Influence of epitope and paratope RMSD on docking quality

In this section we continue the discussion on the importance of epitope and paratope RMSD on docking quality that was begun in the main text. There it was shown that there was a strong correlation between the maximum DockQ score for the top 10 poses and the interface RMSD of the modelled and true antibodies and antigens. We now discuss whether the epitope or paratope RMSD influence docking quality when considered separately.

In Supplementary Fig. 5 and 6, we plot the maximum DockQ score found for the top 10 ranked poses (DockQ-T10) against either the paratope or epitope RMSD after rigid-body and refinement stages, respectively. We further distinguish between different antibody prediction tool. We show both the CDR-Vague-Epi-AA and Para-Epi scenarios, both times using the AlphaFold2 modelled antigen. When comparing the Pearson correlation coefficient ρ, in the CDR-VagueEpi-AA scenario it can be seen that the paratope RMSD is a stronger predictor of the maximum DockQ score than the epitope RMSD across all antibody prediction models. The epitope RMSD has a weak anti-correlation with respect to the maximum DockQ score.

We next evaluate the Para-Epi scenario. In this scenario the paratope RMSD has a stronger anti-correlation to the DockQ-T10 score than the epitope RMSD for ABodyBuilder2 and AlphaFold2, whereas for ABlooper this is reversed. For IgFold, the paratope RMSD has a stronger anti-correlation than the epitope RMSD after the refinement stage, whereas they are comparable after the rigid-body stage.

To further evaluate the impact of the epitope RMSD on the docking success rate, we now divide our 82 antigen structures in 3 categories, based on the corresponding value of the AlphaFold2 antigen epitope RMSD:

- 72 "high-quality" structures have a an epitope showing an RMSD lower than 2.0 Å;
- 9 "good" antigens possess values of RMSD between 2.0 and 5.0 Å;
- 1 "bad" antigen (7LFB) has an incorrectly modelled epitope (RMSD > 5.0 Å) showing an epitope RMSD of 5.95 Å.

We note that HADDOCK3 is never able to generate a good docking pose using the AlphaFold2-modelled antigen structure for 7LFB. This is understandable, as the epitope has been inaccurately predicted by Alphafold2. Supplementary Fig. 7(a-b) shows the average HADDOCK3 SR for top 1 and top 10 models for each protocol over the datasets of good and high quality epitopes. Performances over the 72 high-quality epitopes are typically much better than those on the 9 good quality epitopes, especially for the top 1 structures. For example, in Supplementary Fig. 7a) we can observe how the TOP1 SR of ABBE approaches 60% over the high-quality epitopes.

The trend is still present after flexible refinement and energy minimization (Supplementary Fig. 7(c-d)). When focusing on high-quality epitopes (Supplementary Fig. 7(c)) most protocol achieves a T1-acc SR > 0.60 in the Para-Epi scenario.

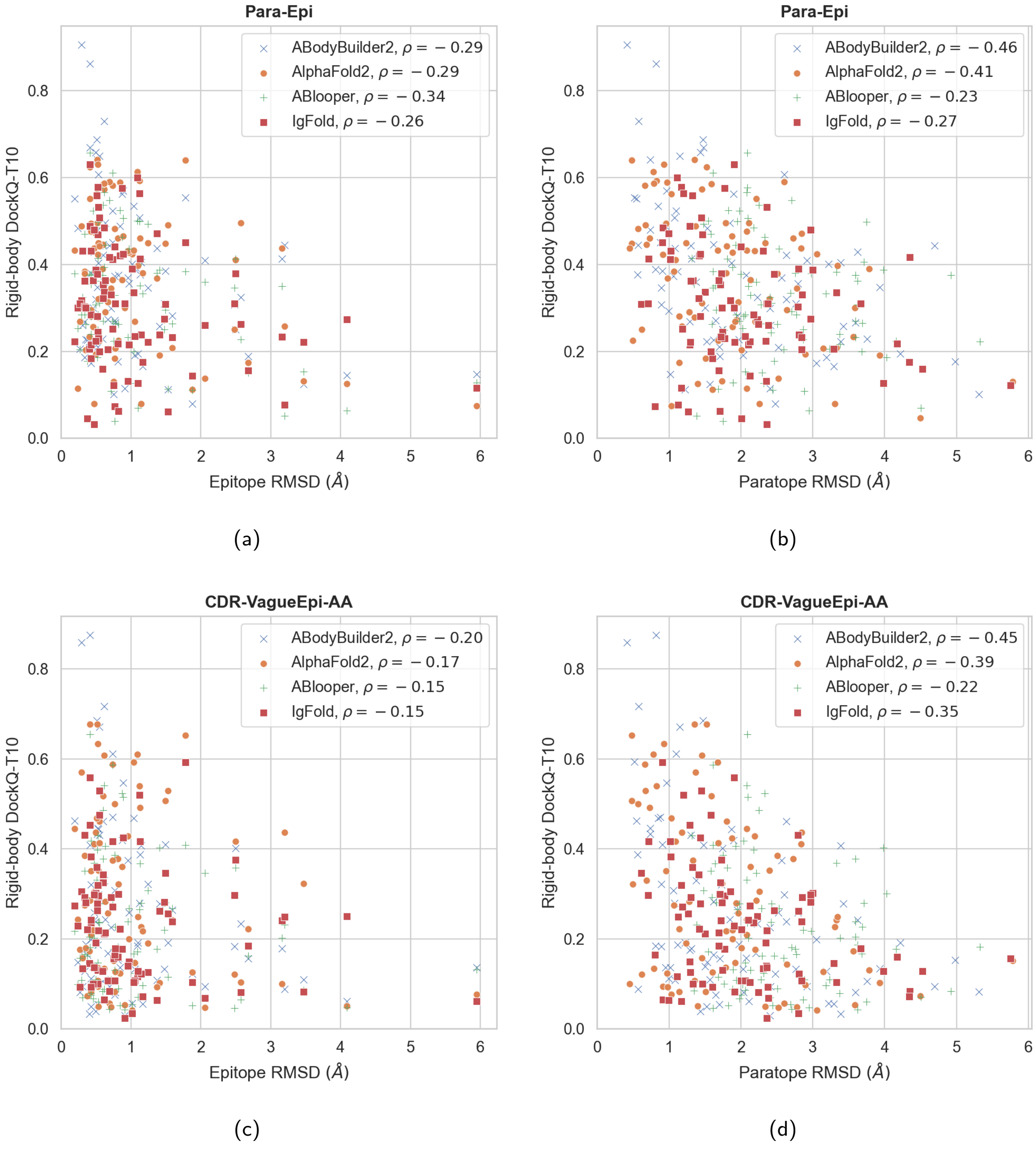

Supplementary Figure 5: Panels (a) and (c) are scatter plots showing the maximum DockQ score in the top 10 ranked poses against the epitope RMSD. Panels (b) and (d) show the maximum DockQ score against paratope RMSD. The models are here taken after the rigid-body stage. Points with different colors and shapes correspond to different antibody prediction tools. All plots use the AlphaFold2 modelled antigen. The Pearson correlation coefficient ρ obtained between the two measurements in each plot is given in the legend, broken down by prediction tool.

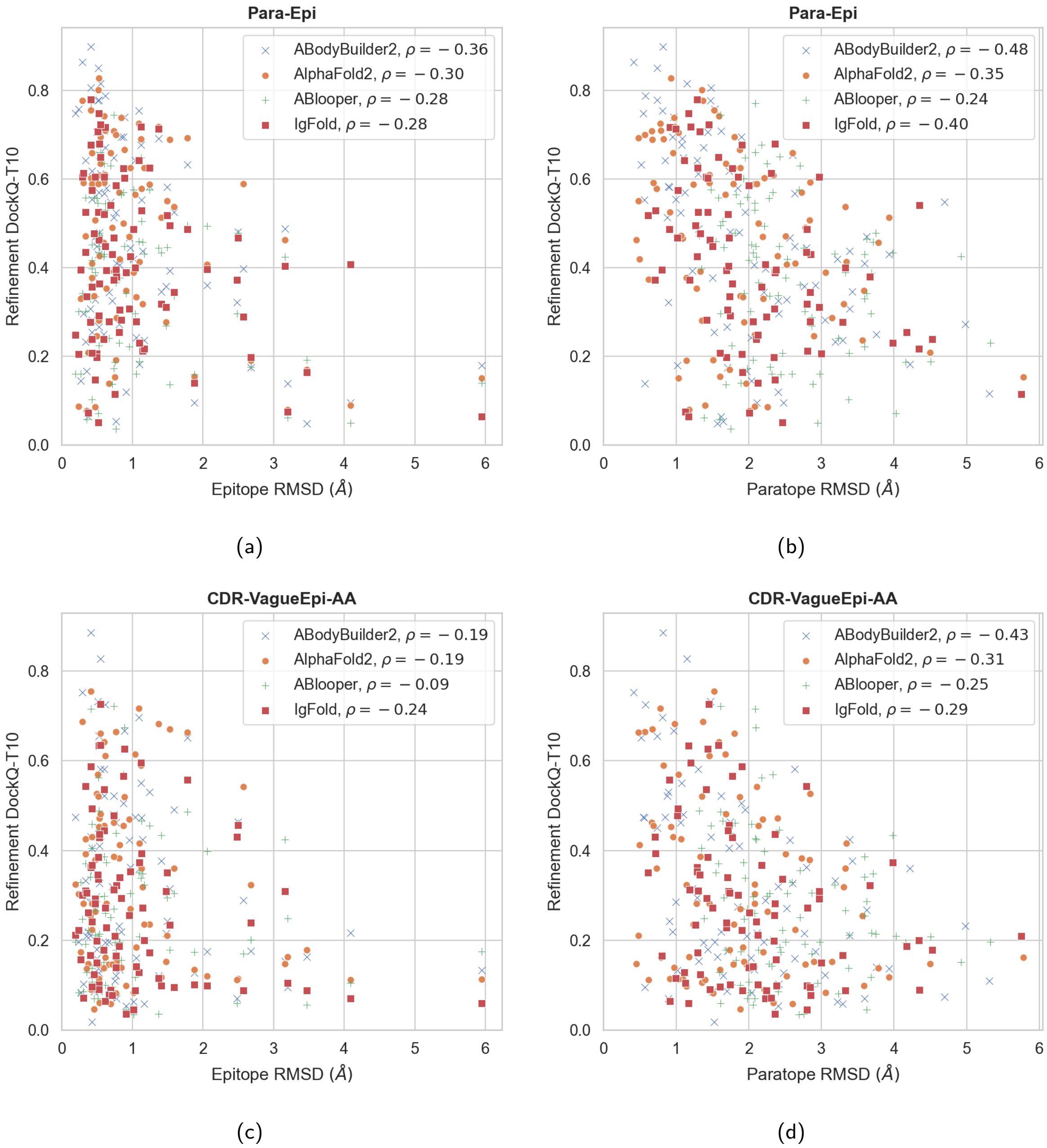

Supplementary Figure 6: Panels (a) and (c) are scatter plots showing the maximum DockQ score in the top 10 ranked poses against the epitope RMSD. Panels (b) and (d) show the maximum DockQ score against paratope RMSD. The models are here taken after the refinement stage. Points with different colors and shapes correspond to different antibody prediction tools. All plots use the AlphaFold2 modelled antigen. The Pearson correlation coefficient ρ obtained between the two measurements in each plot is given in the legend, broken down by prediction tool.

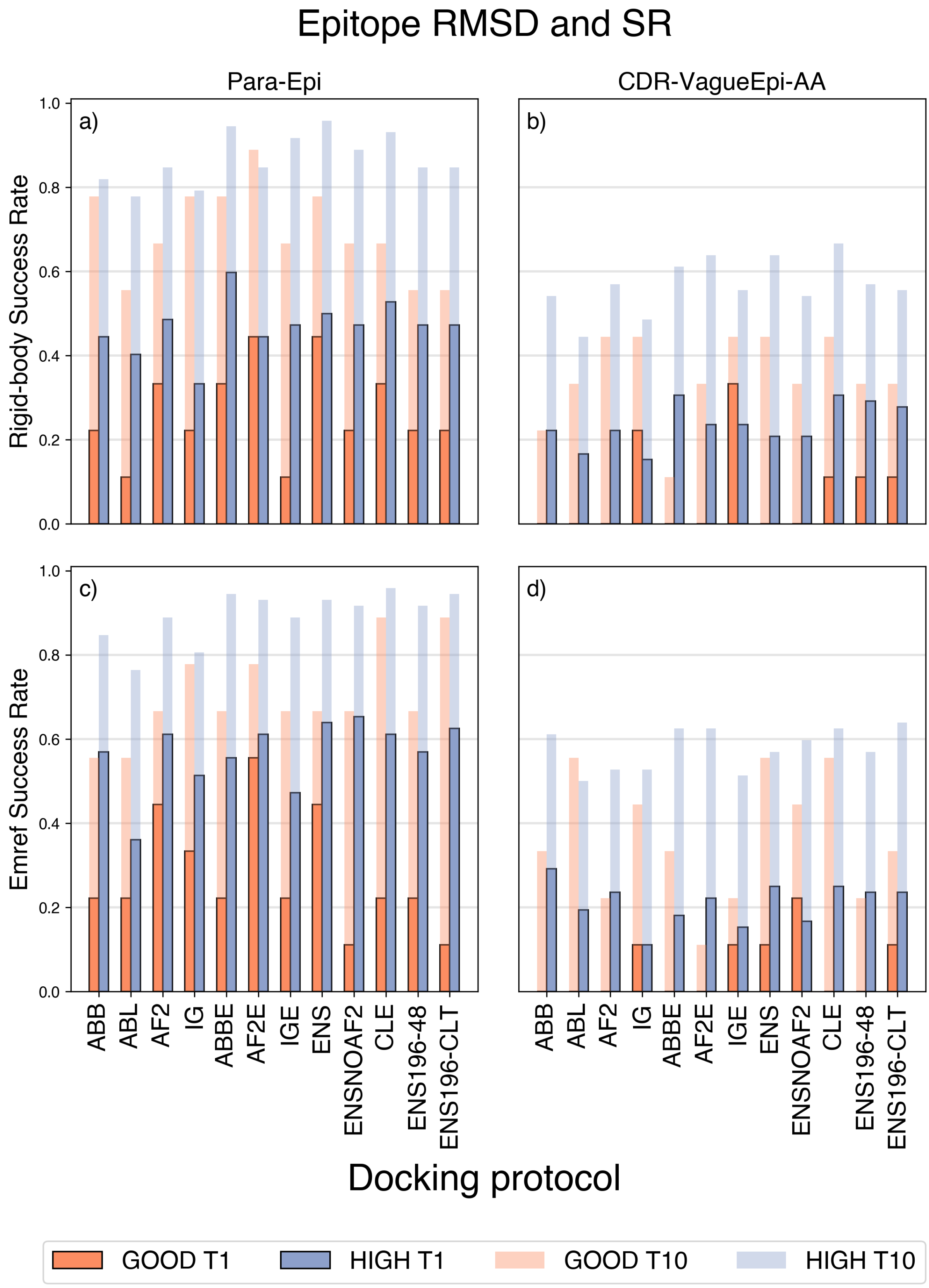

Supplementary Figure 7: Relationship between the epitope quality of the AlphaFold2-modelled antigen and docking success rate for the rigid-body and emref stages of the HADDOCK workflow. GOOD T1 and GOOD T10 bars refer to the success rates over the 9 "good" epitopes (2.0 Å $<$ RMSD $<$ 5.0 Å) the top 1 and top 10 models, respectively. Analogously, HIGH T1 and HIGH T10 bars refer to the docking success rates for the 72 AlphaFold2-antigens with high quality epitopes (RMSD $<$ 2.0 Å). SR values are consistently higher for those runs dealing with a high-quality epitope.

5 Epitope and paratope confidence scores as predictors of docking quality

While the previous section showed that the paratope RMSD and epitope RMSD are correlated with the DockQ-T10 score, these measures are only accessible when the true bound structure is known. If this is the case, then there is no reason to use antibody/antigen modelling tools. Some modelling tools have an inbuilt confidence estimator, the pLDDT score for AlphaFold2 (Jumper et al., 2021), and the RMSPE for ABodyBuilder2 (Abanades et al., 2023). In this section we will investigate if these can be used as a predictor of docking quality. If so, these confidence measures could be used to filter out candidate antibodies during an *in silico* virtual screening campaign in order to avoid low quality docked poses.

To begin, we show the correlation between the RMSD of the AlphaFold2 models compared to the true structures and the mean pLDDT, evaluated both on the epitope and on the paratope residues. This is shown in the scatter plots of Supplementary Fig. 8. We find that the mean pLDDT evaluated on the epitope and paratope residues (as defined by the Para-Epi scenario) is strongly anti-correlated with the RMSD on the corresponding region. In Supplementary Fig. 9, we show how removing structures with a mean epitope (paratope) pLDDT lower than a cutoff value modifies the mean epitope (paratope) RMSD over the remaining data set. We also show the mean of the epitope (paratope) RMSD for the removed structures. We can clearly see that the AlphaFold2 pLDDT can be used to remove antibody and antigen structures that are poorly modelled.

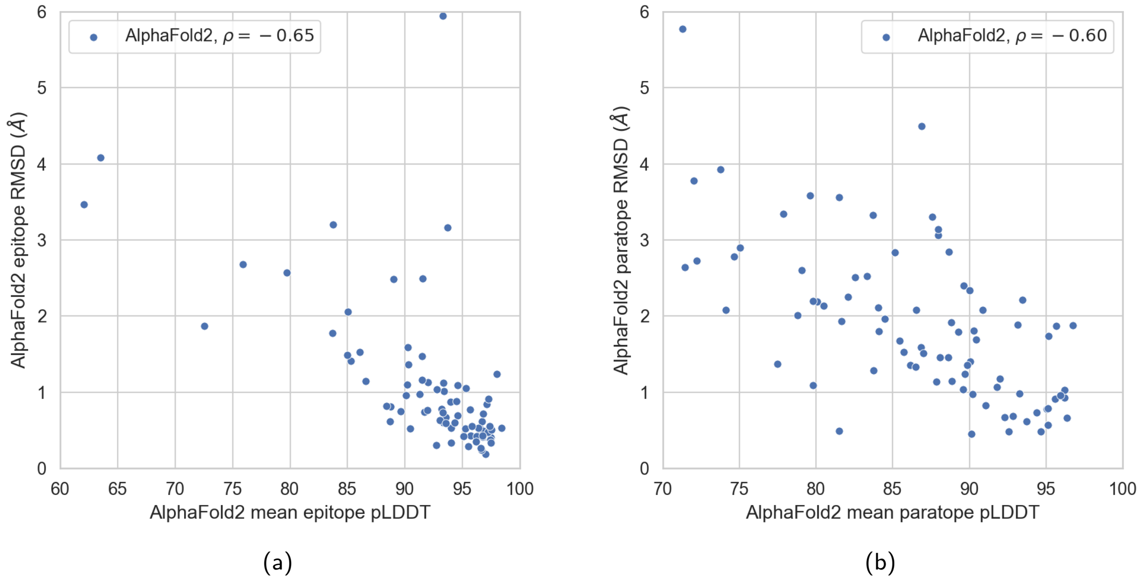

Supplementary Figure 8: Scatter plots showing the correlation between the RMSD and mean pLDDT for AlphaFold2 predicted models for the epitope (left) and paratope (right) residues as defined in the Para-Epi scenario. The epitope RMSD and mean pLDDT have a strong anti-correlation ($\rho = -0.65$) as does the paratope RMSD and mean pLDDT ($\rho = -0.60$), showing that the pLDDT can be used to assess model quality for both regions.

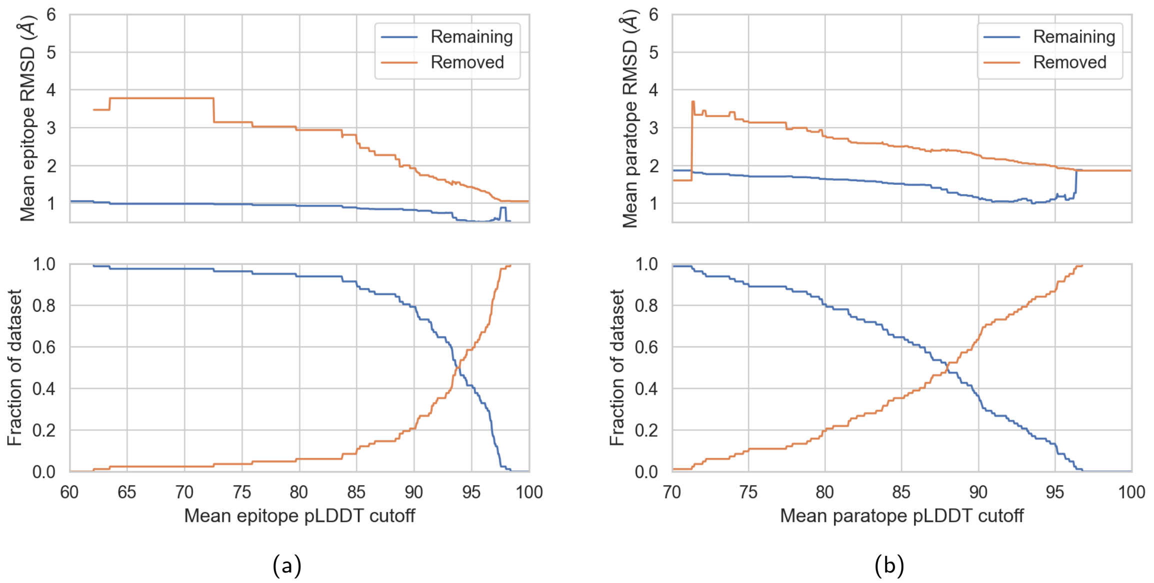

Supplementary Figure 9: Plots showing the effect of filtering out structures with small mean pLDDT values on the mean dataset RMSD. The top plot in each panel shows the mean RMSD for both the removed and remaining structures after filtering out structures with a mean pLDDT smaller than a given cutoff. In the left panel we evaluate the mean pLDDT and RMSD over the epitope residues, and in the right panel we evaluate over the paratope residues. In the bottom plot of both panels we show the fraction of the data set has been removed and how much remains.

Next we evaluate the correlation between the mean ABodyBuilder2 RMSPE for the paratope residues and the paratope RMSD between the true antibody and the ABodyBuilder2 model. We plot this in Supplementary Fig. 10. We find a strong correlation of $\rho = 0.70$, indicating that we can also use the mean RMSPE on the paratope to exclude poorly modelled structures. We confirm this in Supplementary Fig. 11 where we show how removing structures with a mean paratope RMSPE lower than a given cutoff changes the mean paratope RMSD of the remaining data set.

Having established that mean epitope pLDDT and mean paratope pLDDT and RMSPE are correlated with the RMSD across the corresponding regions, we investigate the extent to which they are also related to docking success. We will use the DockQ-T10 values introduced in the previous section.

We first show the correlation between DockQ-T10 and AlphaFold2 mean epitope pLDDT in Supplementary Fig. 12. In the CDR-VagueEpi-AA scenario we present the result after rigid-body docking, and the result after flexible refinement and energy minimisation in the Para-Epi scenario. Notably here we use the CDR-VagueEpi-AA definition of epitope residues to compute the mean pLDDT in that scenario, to simulate the information available in that scenario. We break the correlation coefficients down by the antibody model prediction tool. The mean epitope pLDDT is at best weakly correlated with DockQ-T10 in the CDR-VagueEpi-AA scenario, but that the correlation improves in the Para-Epi scenario. This is particularly true for ABodyBuilder2, which obtains a correlation of $\rho = 0.40$. In Supplementary Fig. 13 we illustrate that for ABodyBuilder2 in the Para-Epi scenario filtering out structures with epitopes that have a mean pLDDT lower than a cutoff can remove docking runs with low DockQ-T10, while keeping runs with high DockQ-T10.

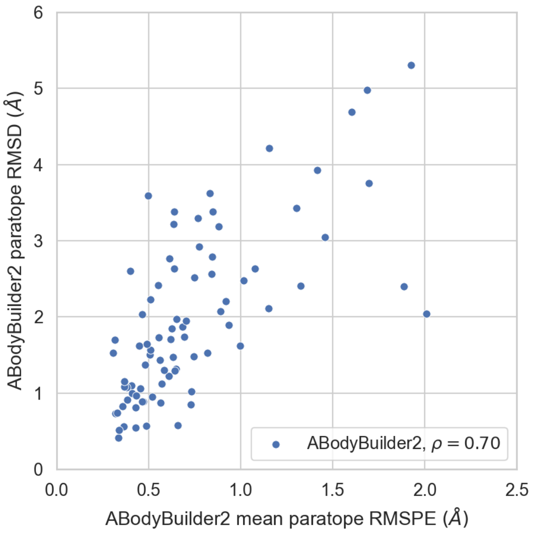

Supplementary Figure 10: Plot showing the relationship between the ABodyBuilder2 mean RMSPE score evaluated on the paratope as defined in the Para-Epi scenario and the RMSD between the ABodyBuilder2 modelled paratope and the true structure. We find a strong correlation of $\rho = 0.70$ between the two variables.

The same analysis can be performed on the mean pLDDT for AlphaFold2 modelled paratopes. In Supplementary Fig. 14 we show how DockQ-T10 is correlated with the AlphaFold2 mean paratope pLDDT after the rigid-body stage for the CDR-VagueEpi-AA scenario, and the flexible refinement and energy minimisation stage in the Para-Epi stage. Again we calculate the mean paratope pLDDT by using the antibody residues specified as the paratope to HADDOCK3 in each scenario. There is a weak correlation $(\rho = 0.21)$ between the two measurements in the CDR-VagueEpi-AA scenario, which slightly increases $(\rho = 0.35)$ in the Para-Epi scenario. In Supplementary Fig. 15 we show that the mean paratope pLDDT can be used to remove antibodies with low DockQ-T10 values after docking in the Para-Epi scenario.

To complete this analysis, we evaluate the relationship between the ABodyBuilder2 mean paratope RMSPE and DockQ-T10. The correlation between these two measurements is shown in Supplementary Fig. 16, where we see that in both the CDR-VagueEpi-AA and Para-Epi scenario we obtain moderate anti-correlation with $\rho = -0.37$ and $\rho = -0.48$ respectively. This is sufficient to filter out antibodies which will obtain poor DockQ-T10 values upon docking with HADDOCK3 in both scenarios, as can be seen in Supplementary Fig. 17.

To summarise, both AlphaFold2 paratope pLDDT as well as ABodyBuilder2 paratope RMSPE show a moderate correlation with docking success and could therefore be used as a pre-screening step in docking algorithms using ML-generated antibody and antigen models. The correlation is stronger in the high information Para-Epi scenario, where more accurate definitions of the paratope and epitope residues are available. For docking runs with ABodyBuilder2 in the Para-Epi scenario, the AlphaFold2 epitope pLDDT can also be used to filter out runs which have low docking success.

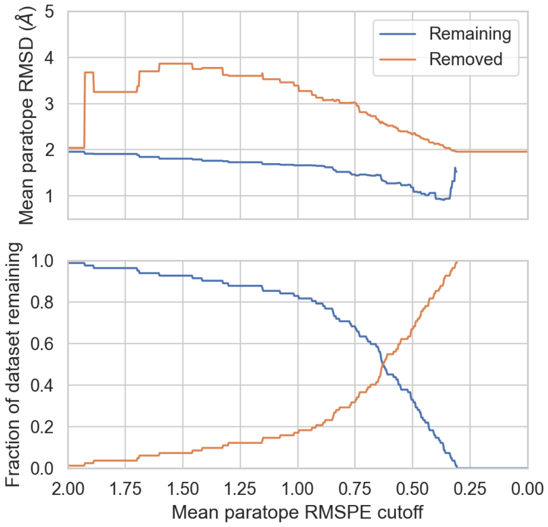

Supplementary Figure 11: Plots showing the effect of filtering out antibodies with large paratope RMSPE on paratope RMSD. The top plot shows the mean paratope RMSD for both the removed and remaining structures after filtering out antibodies with a mean paratope RMSPE larger than the given cutoff. The bottom plot shows the fraction of the data set has been removed and how much remains.

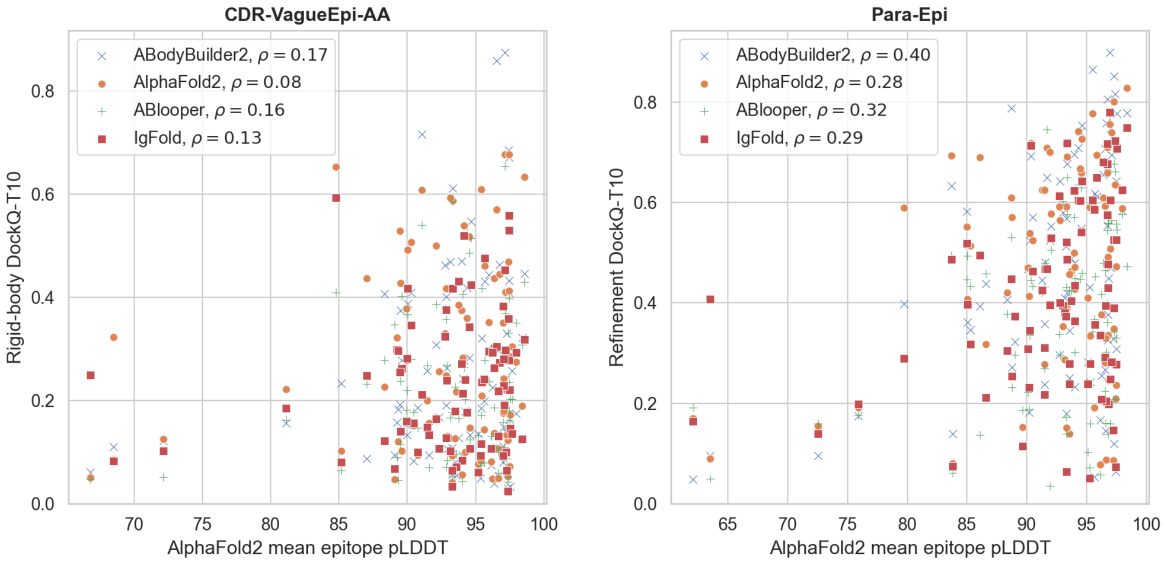

(a) (b)

Supplementary Figure 12: Plots showing the maximum DockQ score in the top 10 ranked poses against the AlphaFold2 epitope pLDDT. Panel (a) corresponds to the CDR-VagueEpi-AA scenario, and (b) to the Para-Epi scenario. The CDR-VagueEpi-AA scenario shows results from the rigid-body stage, whereas Para-Epi shows results after the flexible refinement and energy minimisation stage. To compute the mean epitope pLDDT, the epitope residues are taken to be those as specified to HADDOCK3 in the given scenario. Points with different colors and shapes correspond to different antibody prediction tools. The Pearson correlation coefficient ρ obtained between the two measurements in each plot is given in the legend, broken down by prediction tool.

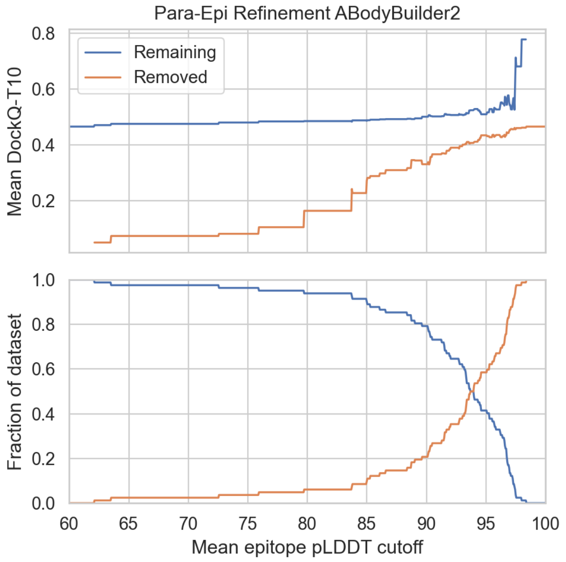

Supplementary Figure 13: Plots showing the effect on DockQ-T10 after filtering out complexes based on epitope pLDDT in the Para-Epi scenario, after flexible refinement and energy minimisation and using the ABodyBuilder2 modelled antibody. The top plot shows the mean DockQ-T10 for both the removed and remaining complexes after filtering out complexes with an antigen with mean epitope pLDDT smaller than the given cutoff. The bottom plot shows the fraction of the data set has been removed and how much remains.

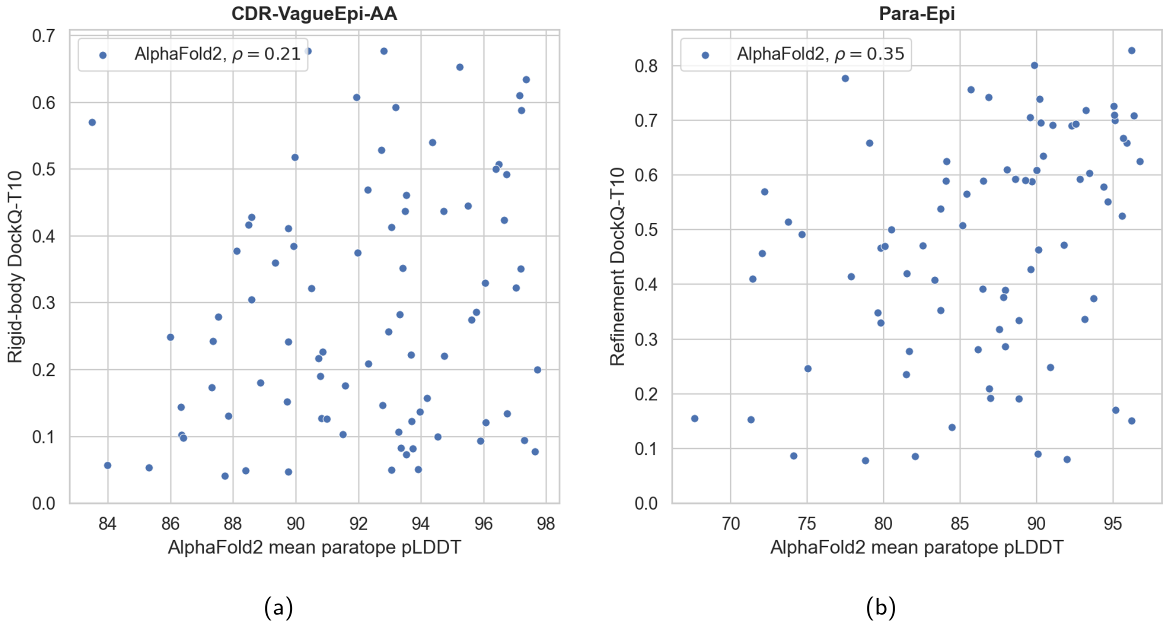

Supplementary Figure 14: Plots showing the maximum DockQ score in the top 10 ranked poses against the AlphaFold2 paratope pLDDT. Panel (a) corresponds to the CDR-VagueEpi-AA scenario, and (b) to the Para-Epi scenario. The CDR-VagueEpi-AA scenario shows results from the rigid-body stage, whereas Para-Epi shows results after the flexible refinement and energy minimisation stage. To compute the mean paratope pLDDT, the paratope residues are taken to be those as specified to HADDOCK3 in the given scenario. Both plots use the AlphaFold2 modelled antigen. The Pearson correlation coefficient ρ obtained between the two measurements in each plot is given in the legend.

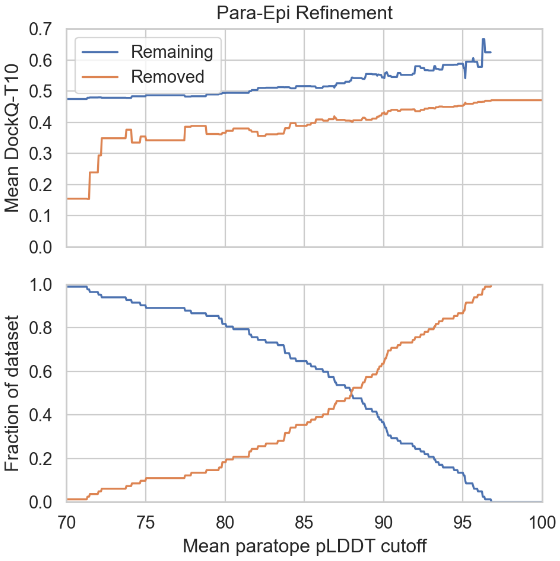

Supplementary Figure 15: Plots showing the effect on DockQ-T10 after filtering out complexes based on paratope pLDDT in the Para-Epi scenario, after flexible refinement and energy minimisation and using the AlphaFold2 modelled antibody. The top plot shows the mean DockQ-T10 for both the removed and remaining complexes after filtering out complexes with an antibody with mean paratope pLDDT smaller than the given cutoff. The bottom plot shows the fraction of the data set has been removed and how much remains.

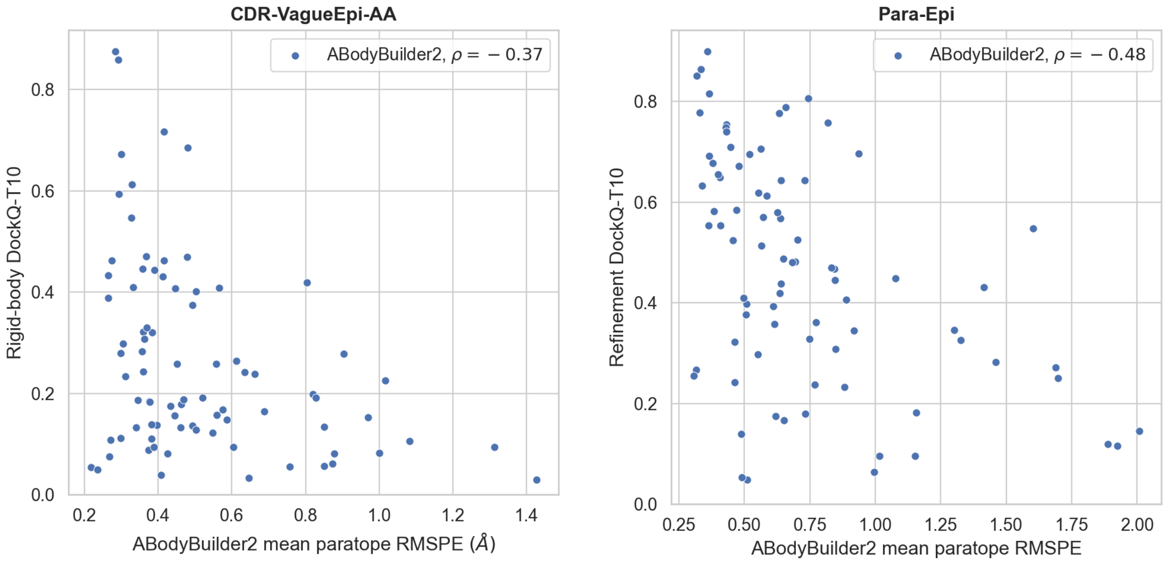

(a) (b)

Supplementary Figure 16: Plots showing DockQ-T10 against the ABodyBuilder2 mean paratope RMSPE. Panel (a) corresponds to the CDR-VagueEpi-AA scenario, and (b) to the Para-Epi scenario. The CDR-VagueEpi-AA scenario shows results from the rigid-body stage, whereas Para-Epi shows results after the flexible refinement and energy minimisation stage. To compute the mean paratope RMSPE, the paratope residues are taken to be those as specified to HADDOCK3 in the given scenario. Both plots use the AlphaFold2 modelled antigen. The Pearson correlation coefficient ρ obtained between the two measurements in each plot is given in the legend.

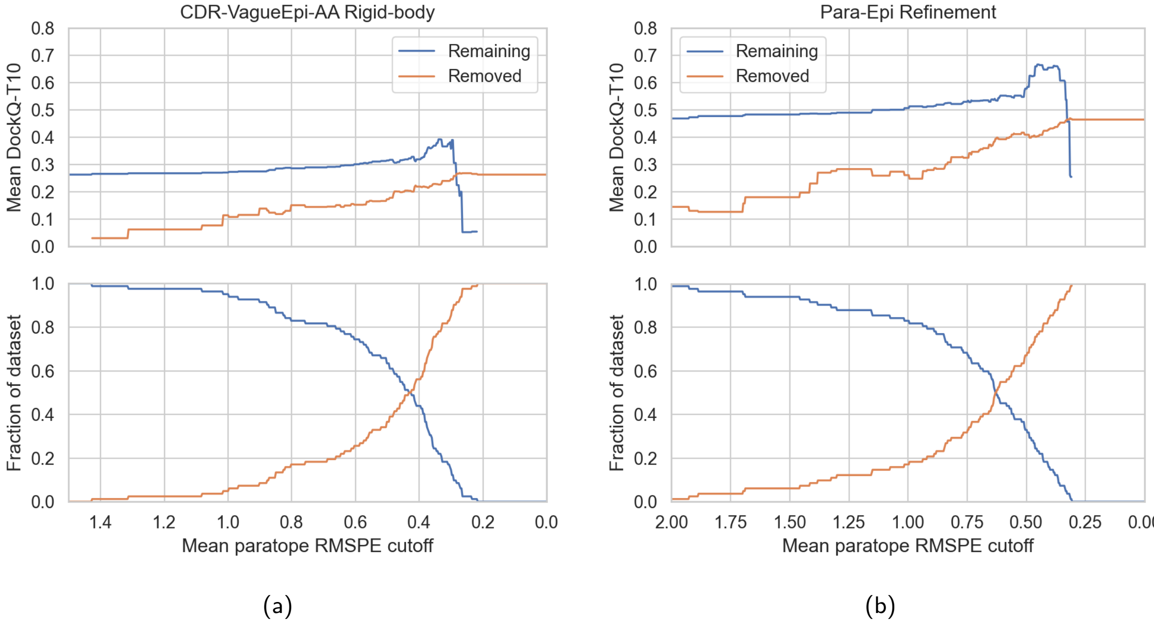

Supplementary Figure 17: Plots showing the effect on DockQ-T10 after filtering out complexes based on mean paratope RMSPE. In both panels the top plot shows the mean DockQ-T10 for both the removed and remaining complexes after filtering out complexes with an antibody with mean paratope RMSPE larger than the given cutoff. The bottom plot shows the fraction of the data set that has been removed and how much remains. The left panel shows the DockQ-T10 results after the rigid-body stage in the CDR-VagueEpi-AA scenario, whereas the right panel shows the results after the flexible refinement and energy minimisation stage after the Para-Epi scenario. All plots use the AlphaFold2 modelled antigen. The residues used to calculate the mean paratope RMSPE in all plots are those specified as the paratope in the given scenario to HADDOCK3.

6 Energy minimisation of rigid-body solutions increases the top 1 success rate

In the main text we discuss the importance of interface refinement for the analysed protocols, highlighting how this CPU-intensive stage can be avoided, especially in the CDR-VagueEpi-AA scenario.

We then applied a short, unexpensive (see Table 2), HADDOCK energy minimisation step to the rigid-body docking models. The results suggest that this energy minimisation is beneficial for the T1-acc SR (see Supplementary Fig. 18), especially when considering run targeting an Alphafold2-modelled antigen, while it seems to have a slightly negative effect on the T10-acc SR, in particular in the CDR-VagueEpi-AA scenario. ABBE, ENS, CLE, ENS196-48, ENS196-CLT protocols show substantial (higher than 0.05) improvements in success rates in the Para-Epi scenario, notably reaching a value of T1-acc around 0.70 without any explicit refinement.

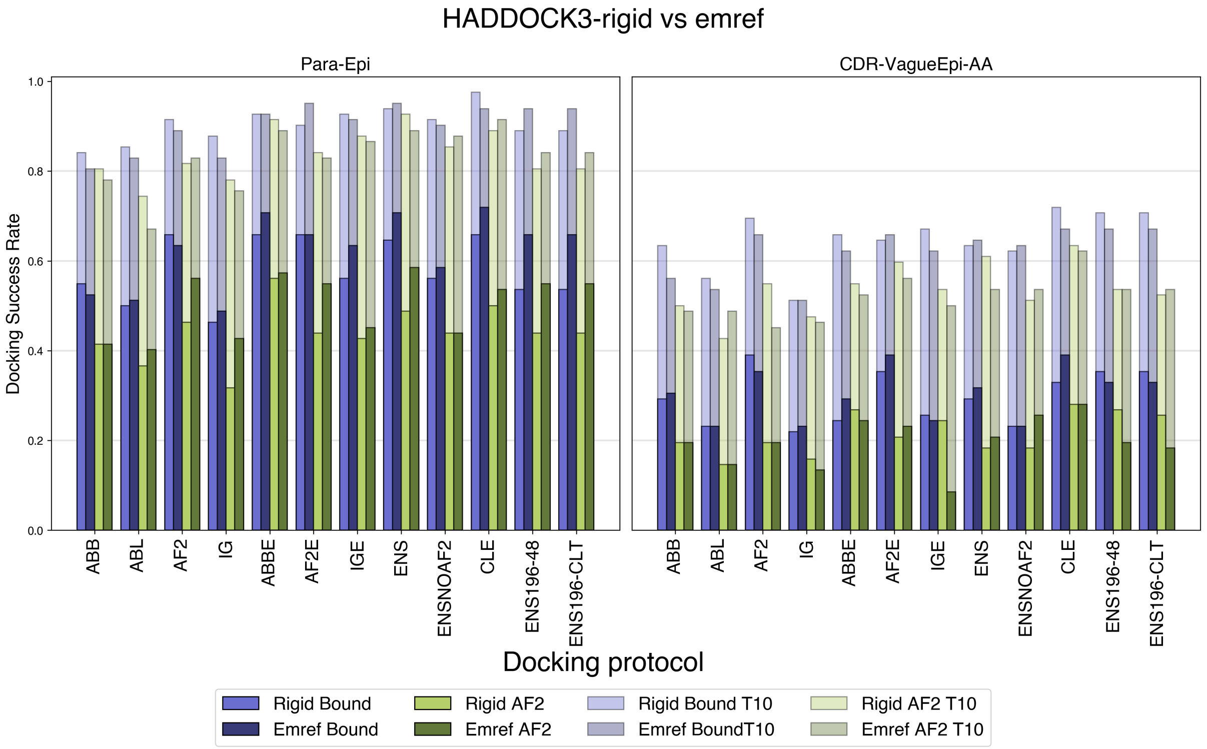

Supplementary Figure 18: Comparison of success rates between rigid-body docking models (RIGID) and energy-minimised rigid-body models (EMREF).

7 Protocol timings

Alongside accuracy, for high-throughput docking applications execution speed is also an important performance metric of docking algorithms. Table 2 shows the average execution time (in CPU hours) for the rigid-body and flexible refinement stages of the HADDOCK3 protocol, alongside the average HADDOCK3 and ZDOCK execution time. The pure rigid-body, FFT-based ZDOCK is faster than HADDOCK3 when considering total execution time. Considering only rigid-body energy minimisation in the Para-Epi scenario however, the two software show similar execution speed. In the main text, we show that HADDOCK3 already produces accurate predictions at this stage, potentially eliminating the need for the resource intensive flexible refinement. It is important to note how HADDOCK3 was run in parallel using 12 cores for all the workflows discussed in the text: the real execution time of each stage can be calculated dividing the CPU time by 12. Therefore, the HADDOCK3 rigid-body stage had an average execution time of 0.027 hours. Another relevant aspect to consider is the impact of the restraints on HADDOCK3 execution time: when accurate information is available (Para-Epi scenario), few restraints are imposed and the minimisation is fast. CDR-VagueEpi-AA runs on the other hand are slower than CDR-VagueEpi runs, as defining two sets of residues as active implies imposing a higher number of restraints.

Supplementary Table 2: Average execution time (in CPU hours) for Default docking protocols (excluding ENS-196-48 and ENS-196-CLT).

Software	Para-Epi	CDR-VagueEpi	CDR-VagueEpi-AA
**ZDOCK**	0.24	0.25	0.25
**HADDOCK rigid**	0.33	0.63	1.09
**HADDOCK flexref**	2.47	3.98	6.04
**HADDOCK emref**	0.20	0.30	0.43
**HADDOCK full**	3.12	5.02	7.67

References

Brennan Abanades, Wing Ki Wong, Fergus Boyles, Guy Georges, Alexander Bujotzek, and Charlotte M Deane. Immunebuilder: Deep-learning models for predicting the structures of immune proteins. *Communications Biology*, 6(1):575, 2023.

Francesco Ambrosetti, Brian Jiménez-García, Jorge Roel-Touris, and Alexandre MJJ Bonvin. Modeling antibody-antigen complexes by information-driven docking. *Structure*, 28(1):119–129, 2020.

John Jumper, Richard Evans, Alexander Pritzel, Tim Green, Michael Figurnov, Olaf Ronneberger, Kathryn Tunyasuvunakool, Russ Bates, Augustin Žídek, Anna Potapenko, Alex Bridgland, Clemens Meyer, Simon A. A. Kohl, Andrew J. Ballard, Andrew Cowie, Bernardino Romera-Paredes, Stanislav Nikolov, Rishub Jain, Jonas Adler, Trevor Back, Stig Petersen, David Reiman, Ellen Clancy, Michal Zielinski, Martin Steinegger, Michalina Pacholska, Tamas Berghammer, Sebastian Bodenstein, David Silver, Oriol Vinyals, Andrew W. Senior, Koray Kavukcuoglu, Pushmeet Kohli, and Demis Hassabis. Highly accurate protein structure prediction with AlphaFold. *Nature*, 596(7873):583–589, 8 2021. ISSN 0028-0836. doi: 10.1038/s41586-021-03819-2. URL `https://www.nature.com/articles/s41586-021-03819-2`.

João PGLM Rodrigues, Mikaël Trellet, Christophe Schmitz, Panagiotis Kastritis, Ezgi Karaca, Adrien SJ Melquiond, and Alexandre MJJ Bonvin. Clustering biomolecular complexes by residue contacts similarity. *Proteins: Structure, Function, and Bioinformatics*, 80(7):1810–1817, 2012.
